# A Review of Analytical Techniques and Their Application in Disease Diagnosis in Breathomics and Salivaomics Research

**DOI:** 10.3390/ijms18010024

**Published:** 2016-12-23

**Authors:** David J. Beale, Oliver A. H. Jones, Avinash V. Karpe, Saravanan Dayalan, Ding Yuan Oh, Konstantinos A. Kouremenos, Warish Ahmed, Enzo A. Palombo

**Affiliations:** 1 Commonwealth Scientific & Industrial Research Organization (CSIRO), Land & Water, P.O. Box 2583, Brisbane, QLD 4001, Australia; akarpe@swin.edu.au or avinash.karpe@csiro.au (A.V.K.); warish.ahmed@csiro.au (W.A.); 2 Australian Centre for Research on Separation Science, School of Science, RMIT University, P.O. Box 2547, Melbourne, VIC 3001, Australia; oliver.jones@rmit.edu.au; 3 Department of Chemistry and Biotechnology, Swinburne University of Technology, P.O. Box 218, Hawthorn, VIC 3122, Australia; epalombo@swin.edu.au; 4 Metabolomics Australia, Bio21 Molecular Science and Biotechnology Institute, University of Melbourne, 30 Flemington Road, Parkville, VIC 3010, Australia; sdayalan@unimelb.edu.au (S.D.); konstantinos.kouremenos@unimelb.edu.au (K.A.K.); 5 WHO Collaborating Centre for Reference and Research on Influenza (VIDRL), Peter Doherty Institute for Infection and Immunity, 792 Elizabeth Street, Melbourne, VIC 3000, Australia; DingThomas.Oh@influenzacentre.org; 6 School of Applied and Biomedical Sciences, Federation University, Churchill, VIC 3350, Australia

**Keywords:** breath, saliva, metabolomics, bioinformatics, gas chromatography, liquid chromatography, mass spectrometry

## Abstract

The application of metabolomics to biological samples has been a key focus in systems biology research, which is aimed at the development of rapid diagnostic methods and the creation of personalized medicine. More recently, there has been a strong focus towards this approach applied to non-invasively acquired samples, such as saliva and exhaled breath. The analysis of these biological samples, in conjunction with other sample types and traditional diagnostic tests, has resulted in faster and more reliable characterization of a range of health disorders and diseases. As the sampling process involved in collecting exhaled breath and saliva is non-intrusive as well as comparatively low-cost and uses a series of widely accepted methods, it provides researchers with easy access to the metabolites secreted by the human body. Owing to its accuracy and rapid nature, metabolomic analysis of saliva and breath (known as salivaomics and breathomics, respectively) is a rapidly growing field and has shown potential to be effective in detecting and diagnosing the early stages of numerous diseases and infections in preclinical studies. This review discusses the various collection and analyses methods currently applied in two of the least used non-invasive sample types in metabolomics, specifically their application in salivaomics and breathomics research. Some of the salient research completed in this field to date is also assessed and discussed in order to provide a basis to advocate their use and possible future scientific directions.

## 1. Introduction

Recent advances in analytical technologies and high-throughput data analysis techniques have established metabolomics as one of the emerging ‘*omics*’ platforms within systems biology research [1], where ‘*omics*’ comprise genomics, transcriptomics, proteomics and metabolomics [2]. For the purpose of this review, metabolomics is defined as the study of all the small (<1000 amu) chemical compounds that are either produced or consumed within a biological system. If done correctly, metabolomics can provide an unbiased analysis of all such small-molecule metabolites (collectively known as the metabolome) within a given biological system, under a given set of conditions, through the analysis of a variety of different and complementary samples [3]. As such, metabolomics has the potential to improve disease diagnosis as well as the characterization of underlying pathological mechanisms, and more broadly, monitoring and understanding phenotypic variation; a clear differentiator from genomic and transcriptomic based approaches [2].

A recent review by Bujak et al. [1] of metabolomics-based diagnostics highlighted that saliva and breath analysis currently constitute only a small fraction of the total number of sample matrices investigated in metabolomics research. The authors reported that between 2010 and 2015 saliva and breath samples were the subjects of only circa (ca.) 6.0% (4.6% saliva based and 1.4% exhaled breath based) of metabolomics research, with urine (21.3%), blood (plasma/serum) (68.5%), and tissue homogenates (4.4%) constituting the remaining sample types studied.

Collection methods for such metabolomics samples are generally either considered invasive (such as blood and tissues) or non-invasive (such as saliva, breath and urine). The cost of sampling and analyzing invasive samples can be relatively high and the collection of some sample types, such as urine (and feces), can be socially awkward or embarrassing for the patient. To eliminate such issues, the collection of non-invasive samples such as exhaled breath or saliva are increasingly being considered. For example, while urine is considered a non-invasive sample matrix, aside from the social considerations (i.e., being awkward or embarrassing for the patient to collect), it still poses some issues in terms of sample stability. In addition, the focus of urine-related metabolomics has been well documented, and the interested reader is directed to the recent reviews on urine-based metabolomics [4,5].

Saliva and breath sampling methods are considered to be truly non-invasive and can be performed easily without any embarrassment or discomfort [6]. Furthermore, both have existing sets of predefined sample collection protocols and tools that facilitate reproducible sampling and patient self-sampling (if required). As such, the purpose of this review is not to provide a comprehensive commentary on salivaomics and breathomics; both have been covered in numerous independent reviews already [7,8,9,10]. Instead, the focus of this review is to highlight the benefits of two of the least used non-invasive sample types (i.e., saliva and breath) in metabolomics research. To do so, a summary of the sampling tools, protocols and approaches, and the analytical platforms used to analyze these non-invasive sample types, is presented. Lastly, a selection of saliva and breath case study applications is used to inform a discussion on the future direction of salivaomics and breathomics, and to advocate their increased use as a diagnostic medium for use in personalized medicine.

## 2. Breath and Saliva Sampling Considerations

With the advancement of metabolomics technology, saliva and exhaled breath can be leveraged as a clinical and diagnostic tool due to their potential to mirror oral and respiratory health, as well as other systemic health conditions within the human body [11]. To the layperson, saliva appears to be a homogenous fluid. However, saliva is in fact a mixture of different fluids made from three distinct salivary glands (namely, the parotid, the submandibular, and the sublingual glands). The three glands contain common compounds (i.e., cortisol, α-amylase, and secretory immunoglobulin A (SIgA), to name a few) but the concentrations of these substances can vary significantly depending on the gland(s) sampled. For example, the parotid glands produce a watery type of saliva comprised of large amounts of digestive enzyme α-amylase and proline-rich proteins, and low levels of compounds such as cystatins and histatins. Whereas, the sublingual glands produce a viscous saliva fluid with high concentrations of glycoproteins and the enzyme lysozyme. Submandibular glands, on the other hand, produce a mixture of both saliva types which typically contains a high concentration of cystatin C and are also comprised α-amylase (albeit at a reduced level compared to parotid gland saliva) [12,13]. A small amount of saliva is also secreted through hundreds of minor glands located within the mouth (i.e., lips, tongue, palate, and cheeks). As such, when sampling saliva, it is vital that the saliva collected is characterized in terms of its location, and the variation of these saliva biomarkers are first determined and used to normalize sample sets in any resulting omics-based studies [12,13]. For this reason, most studies report assisted and self-sampling approaches that collect the whole saliva mixture comprised of all the various glandular saliva types produced in the mouth. While the protein composition arising from each salivary gland is well characterized, the composition of metabolites is not well understood. However, it has been determined that saliva comprises more than 800 identified metabolites, ranging from carboxylic acids and derivatives, steroids and steroid derivatives, to quaternary ammonium salts and polyketides, and appears to be comparable to the human serum and cerebrospinal fluid metabolomes in terms of chemical complexity and metabolite abundance [7].

It is of note that such a sample may also contain other biological fluids such as tears, nasal and bronchial secretions, bacteria (and products derived from them), as well as blood products (from mouth micro-injuries or oral disease). As such, the composition of saliva is not constant, and it changes depending on the degree of stimulation to the glands, time of day, diet as well as health status. This reinforces the fact that at the point of sample collection all supporting metadata are captured and standardized [14,15]. As a general guide when undertaking a biomarker discovery study, samples should not be collected within 45 min of brushing of teeth [16], within 24 h of dental work being performed and/or when there is evidence of oral health issues or injuries, or when contaminated with blood [16,17]; such contaminated samples should be discarded. Granger et al. [18] also advise that the use of stimulants should be avoided, as they can introduce bias in terms on analyte concentrations and dilutions. Furthermore, prior to sampling, subjects should avoid sugary or acidic foods as they have been observed to lower saliva pH and increase bacterial growth [19,20]. Alcohol, caffeine, nicotine, and prescription/over-the-counter medications should also be avoided (for around 12 h) prior to sampling to avoid biasing any resulting data [19,21].

Breath, like saliva, offers an attractive sample alternative to blood; it is relatively simple and non-invasive to collect [22]. Breath sampling methods must however, take into consideration the diffusion of volatile organic compounds (VOCs) from blood to alveolar air (where the alveolar breath is the ca. 350 mL end portion of a breath), which depend on polarity, Henry’s law partition constants (air-water partition coefficients), solubility in fat and volatility [23]. Cao and Duan [24] proposed the calculation of partition coefficients (and factors affecting these coefficients which should be taken into account) for all compounds measured in breath. In addition, when undertaking a biomarker discovery study using breath, care should be taken to maximize sample quality by minimizing interferences from exogenous environmental VOCs (e.g., by minimizing contamination from surroundings, nasal passages and the oral cavity itself) or VOCs that do not originate from the source of interest [25]. 

Although the above is indeed recommended, the authors also propose that the sampling process should also aim to be as chemically unselective as possible. However, it should be noted that once a biomarker has been identified in either saliva or breath, the merit, uptake and success of these tests within a clinical diagnostic setting would ultimately rely on less stringent sampling constraints for patients (i.e., removing such restrictions as teeth brushing, dental work, food etc.). Such sampling restrictions and caveats are considered undesirable in rapid diagnostic tools.

In these types of studies, it is important that both biological and analytical precision (or repeatability) are first determined and subsequently reported. This is typically done through reporting the intra- and inter-Coefficients of Variability (CV), which account for biological and analytical variations within the sampled cohort. Furthermore, in larger studies with many samples to be tested, it is necessary that samples be analyzed in batches, each with its own independent set of calibrators for the standard curve, and a series of saliva/breath controls with known concentrations of standards and surrogates. Most studies measure each sample in duplicate or triplicate for each analyte, with some researchers extending beyond this and analyzing each sample up to 10 times (more in some cases) [26]. For saliva and breath condensate, pooled samples are typically used as a quality control measure [27,28]. Such a protocol ensures that both sample variation and analytical drift are captured and corrected, ensuring that the analyzed breath and saliva samples are scientifically valid.

Furthermore, it should be noted that one of the major pitfalls in metabolomics research, as is the case in biological research in general, is the inability to reproduce results of published studies. This was discussed in detail in two well-publicized papers by Ionnidis [29] and Button et al. [30]. The conclusions drawn in these papers for the inability to reproduce results in biological sciences applies equally well to salivaomics and breathomics studies. The major reasons identified by Ionnidis [29] and Button et al. [30] were: low power studies (i.e., experiments that are designed with a small sample size) and an observed smaller effect size between the compared groups (i.e., the statistical significance that defines the difference between groups is close to the 0.05 *p*-value cutoff). In addition to the above, an added complication in omics-based research involving humans are the substantial subject-to-subject variation individuals’ exhibit. This is specifically pertinent while analyzing breath and saliva samples. Thus, one of the methods of obtaining statistically significant results that are reproducible is to have studies that have a large sample size, attempt to reduce the variation between subjects within a group and to focus on findings that statistically show a substantial difference between two or more compared groups.

## 3. Sampling Techniques

The following section aims to provide information relating to saliva and breath sampling techniques and summarize the approaches and technologies that can be used in salivaomics and breathomics research.

The primary functions of saliva include facilitating digestion, assisting in swallowing and providing a lubricant and protective barrier against harmful bacteria and pathogens within the buccal (oral) cavity [31]. The salivary glands are highly permeable, with each gland being wrapped by capillaries that facilitate the free exchange of blood-based molecules into the saliva-producing acinus cells [8]. As such, it is thought that saliva may contain molecular information relating to an individual’s general current health status [8]. Exhaled breath is another biological medium that potentially contains molecular information relating to health. For this to be true, the relevant metabolites need to freely transfer from the blood stream into the air within the gas exchanging region of the lungs via the alveolar–capillary barrier [32]. It is important to note, that VOCs also originate directly from local cellular metabolism within the airways [33]. With that in mind, the following sections details common sampling approaches for saliva and breath that are frequently used in metabolomics analyses focused on biomarker discovery for disease diagnostics.

### 3.1. Saliva Sampling

The origins of saliva sampling methods can be traced back to the work of May et al. [34] who were the first to introduce the use of a dental cotton roll to collect saliva to monitor the drug desipramine [34]. Some improvements have been made to their method, and since 1990, saliva collection kits have been available commercially as the Salivette^®^ cotton swab (Sarstedt AG & Co., Nümbrecht, Germany). The Salivette^®^ is placed in the mouth and chewed for 30–45 s and then placed in a container which is closed with a stopper. The container (in a polystyrol tube) is then centrifuged for 3 min at 1000× *g*. The saliva passes into the lower end of the tube and is collected. A known disadvantage associated with the Salivette^®^ collection approach is that there could be an over-stimulation of saliva glands that result observed in interference caused by the dental roll with some hormone and drug assays (e.g., for testosterone) being over or under represented due to Salivette^®^ roll stimulating some saliva glands [34].

Recent sampling methods for saliva metabolomics have however, evolved from the saliva preparation method proposed by Sugimoto et al. [35], which in turn was based on the salivary transcriptome work of Li et al. [36]. Briefly, subjects are asked to fast for at least 1 h prior to saliva collection. Mouths are first rinsed with water and saliva samples are collected 5 min later into 50 cc Falcon tubes immersed in crushed ice. A total volume of 5 mL of saliva can typically be collected within 10 min with this method. Such saliva samples are immediately centrifuged at 2600× *g* for around 30 min at 4 °C. The supernatant is then transferred to fresh tubes, with samples frozen at −80 °C within 30 min.

Dame et al. [7] used a similar method for collection of saliva, where subjects spat into a 50 cc sterile Falcon tube for 2–3 min, with a 2.5 mL total volume collected. Samples were collected three times a day (before breakfast, 2 h after breakfast and 2 h after lunch), in order to account for sampling time and diurnal variations. Following collection, the samples were centrifuged at 10,000× *g* for 10 min and aliquots were stored in Eppendorf tubes at −20 °C. The pH of these samples was also measured before analysis, which was found to be within the normal range (7.22 ± 0.64). Methods similar to those described by Dame et al. have been used in several recent studies, thus one could almost say that a “standardized” saliva collection protocol for metabolomics has been established [26,37,38]. However, more work is needed before this statement is stated fully accurate.

The passive drool technique is one of the most widely used methods for saliva sampling. Here, saliva is allowed to pool on the mouth-floor and is then collected in a cryovial by tilting the head. The method is very cost effective, universally approved and easily applied [39]. In cases where passive drool cannot be used, such as infants, children and people with certain disabilities, oral swabs (including those for children and infants) are used. The swab is kept under the tongue or mouth-corners for about 1.0–1.5 min. The saliva sample is then quickly extracted from the swab using centrifugation or compression [39]. Similar swab based methods are used to extract saliva from animals. The methods include cotton ropes or pads (deer, primates etc.), hydrocellulose eye spears (dogs), sponges (pigs, primates) and plastic spoon (rhinoceros) [40].

A recent study aimed to analyze the effects of physiological and environmental parameters on salivary metabolomic profiles, taking into account numerous lifestyles and health related issues. In this study, Sugimoto et al. [41] concluded that collection methods, collection time, patient’s gender, body mass, and their smoking status impacted the metabolomic profile the most. However, parameters such as tooth brushing or the use of medications or nutritional supplements had a minimal affect. In addition, the study indicated a metabolic difference between the sampling methods such as the higher concentrations of aspargine and guanidine (agmatine) by the passive drool method [41]. Similarly, functional magnetic resonance imaging (fMRI) tests indicated a direct proportionality between saliva uric acid content and increased psychosocial stress [42].

A passive drool based targeted approach using ultra-high performance liquid chromatography coupled with ion mobility-mass spectrometry (UHPLC-IM-MS) was later able to identify *δ*-valerolactam as a potential signature metabolite for exercise induced physiological stress on the human body [43]. A more recent, untargeted UHPLC-Time of Flight-MS (UHPLC-TOF-MS) analysis also indicated the presence of about 10 diagnostic biomarkers of asthma. Some of these potential biomarkers, especially those with a lower carbon number (C_5_–C_15_; 100 < *m*/*z* > 450) were observed to be upregulated during the asthma condition compared to the higher molecular weight biomarkers (C_11_–C_28_; based compounds 220 < *m*/*z* > 600) [44].

### 3.2. Breath Sampling

Breath sampling methods range from initial experiments conducted by breathing directly into an analysis platform [45], to more recent collection methods using Tedlar^®^ bags (E. I. du Pont de Nemours and Company, Wilmington, DE, USA), aluminized Mylar bags, Tenax^®^ cartridges (Buchem B.V., Apeldoorn, The Netherlands), Bio-VOC™ bottles (Markes International Ltd., Llantrisant, UK) and more recently the EBC condenser, ReCIVA™ (Owlstone Ltd., Cambridge, UK), RTube™ (Respiratory Research Inc., Austin, TX, USA) and QuinTron™ samplers (QuinTron, Milwaukee, WI, USA). Table 1 lists a range of available breath sampling devices, with an assessment of the advantages and disadvantages associated with each device.

Work from Pleil et al. in 2013 used a device called the “single breath canister”, which comprised of a stainless steel canister with room for 1.0–1.5 L of alveolar breath [25]. The device made use of a Teflon tube as a mouthpiece, with isoprene and acetone being used as markers to assess the quality of exhaled breath. Earlier work by Dyne et al. [46] had proposed a device which was portable and robust and was based on the last part of the breath being held within a tube and sampled. More recently, a commercially available sampling system called the “Bio-VOC™ sampler” was produced by Markes International (Llantrisant, UK), which was based on the pioneering work carried out at the UK Health and Safety Laboratory (Buxton, UK) [47]. In this sampler, only the final part of the breath (ca. 150 mL) is retained and is assumed to come entirely from the alveolar portion of the lungs. The sample can then be pushed onto a sorbent tube (which is then capped), or Tedlar bag through a connection to the opened end of the cylinder.

A range of pre-concentration techniques consisting stainless-steel/glass tubes (containing adsorbents used for various samples) is available for saliva/breath sampling. An adsorbent that is appropriate for all VOCs must be chosen to pack such tubes, and this is not always an easy task. There are four main different types of adsorbents: porous organic polymers, activated charcoal, carbon molecular sieves and graphitized carbon blacks (which can be porous or non-porous). Tenax (2,6-diphenyl-*p*-phenylene oxide) is probably the most commonly used adsorbent due to its hydrophobicity, thermal stability and its ability to absorb a wide range of VOCs.

Some researchers collect exhaled breath condensate (EBC), which is a biofluid obtained non-invasively after collecting and cooling the exhaled air [6,48,49]. Typically, the condensate is collected via a sampling device fitted with a condenser and a saliva trap. A major advantage of analyzing EBC is that it captures both volatile and non-volatile metabolites [50]. The EBC is collected over a period of 15 or more minutes and its composition is believed to reflect that of the fluid lining the airways [48].

One of the recently developed methods, namely exhaled breath vapor/condensate (EBV/EBC) collection, has been described in a widely cited research paper by Marti et al. [51]. The study involved the use of a solid phase microextraction (SPME) fiber fitted inside the commercial breath collection device, namely the RTube™. The SPME adsorbed sample was then desorbed to GC-MS assembly for analysis. The test indicated the presence of limonene and related metabolites such as pinene, myrcene and terpinols from breath samples of individuals who had consumed lemonade and also showed great potential for picking up compounds more relevant to medical diagnosis.

A recent review also indicated the importance of EBV/EBC in conjugation with techniques such as Gas Chromatography Mass Spectrometry (GC-MS), Nuclear Magnetic Resonance (NMR) spectroscopy and sensor systems for analyzing a real time ‘*breathprint*’ for diagnosing respiratory issues such as asthma, lung cancer, cystic fibrosis and chronic obstructive pulmonary disease (COPD) among others [52]. Similar work involved the use of breath VOCs as markers for irritable bowel syndrome (IBS). The experiment in question used Tedlar Bag^®^ bags (E. I. du Pont de Nemours and Company, Wilmington, DE, USA) to collect the breath samples of patients [53]. The breath samples analyzed by thermal desorption-GC-TOF-MS indicated the presence of 16 biomarkers related to IBS, with about 89.4% sensitivity and 73.3% specificity of IBS. Correlation between sets of gastrointestinal symptoms and the VOCs biomarker set for both clinical IBS patients and subjects in a general population cohort were also reported [53].

A similar approach, albeit using extractive electrospray ionization mass spectrometry (EEI-MS), was able to detect and correlate the direct relationship between the creatinine concentration and chronic kidney disease (CKD) [54]. The test, involving 100 volunteers (50 healthy and 50 with CKD), indicated no creatinine in healthy individuals, but detected 42–924 parts per trillion volumes in breath samples of CKD patients.

A similar principle was also successfully utilized to detect and determine signature metabolite markers of *Plasmodium falciparum* infection (causing malaria). Nine signature metabolites were detected, including CO_2_, acetone, isoprene, benzene, cyclohexnone and thioethers [22]. It should be noted however, that these compounds are not rare compounds in breath and an increase in their levels could just as easily be diagnostic of general illness. More recently, a “breathe free” consortium (www.breathe-free.org) has been launched to establish a network of researchers interested in breath analysis, as well as address issues associated with data collation. In addition, the “ReCIVA” (Respiration Collector for In Vitro Analysis; Owlstone Ltd., Cambridge, UK), breath sampler has been developed, and is currently in use in seven hospitals worldwide for the detection of lung cancer. Some of the advantages associated with the ReCIVA sampler include: flexibility that allows a selection of different breath volumes and fractions (alveolar, bronchial) to be collected; reproducible and repeatable sampling; and, potential to store sorbent tubes for later analysis. However, it should be noted that further validation and head-to-head comparisons of the ReCIVA sampler with other devices are needed. Such commercial sampling devices are also designed to minimize VOC carryover or adsorbent memory [55]. It should be noted that breath samples are considered rather stable; short-term storage of 1 month did not affect sample stability, as demonstrated in a urea breath best samples for the diagnosis of *Helicobacter pylori* [56]. Furthermore, Kang and Thomas [57] demonstrated the storage of VOCs trapped from breath samples on a dual bed Tenax^®^ TA:Carbograph adsorbent tube (Buchem B.V., Apeldoorn, The Netherlands) and stored −80 °C. In this study, it was shown that samples produced reproducible data after 1.5 months with 94% stability of the VOCs. However, ca. 30% of VOCs were degraded at after 6 months of storage [57].

Overall, it has been indicated that breath tests are currently the earliest and least invasive of the metabolomic profiling sampling methods. In the case of communicable diseases, further development of this technology may aid in preventing disease spread via enabling faster and earlier diagnosis. 

## 4. Instrumentation and Analytical Techniques

A major challenge in any form of metabolomics is to address the extremely diverse and complex nature of the compounds being analyzed. The term “metabolites” cover a huge range of bio-molecules exhibiting a wide range of concentration levels and physico-chemical characteristics such as mass and polarity. More than 3000 compounds have been detected in breath (although an “average” sample contains only around 200 compounds) [58], while the recently published human saliva metabolome (www.salivametabolome.ca/) identified 853 metabolites.

Unlike blood, urine or cerebrospinal fluid, saliva can not only be easily, non-invasively and repeatedly obtained, it is relatively simple to analyze. Furthermore, some researchers have demonstrated the stability and reproducibly of saliva-based metabolites and proteins in longitudinal studies [59,60]. However, it is not as widely utilized in diagnostic studies as other bio-fluids despite its demonstrated applicability for such purposes [9] even in diseases not related to the pulmonary system [61]. In contrast, breath, whilst also easy to sample, is a particularly challenging sample matrix due to the low concentrations of metabolites within it and the fact that many common analytical methods are not designed for gas analysis; although breath condensate is mostly water, it is the volatile compounds in the gas phase are often the most diagnostically useful.

A variety of methods have been utilized to study saliva and breath ranging from the more commonly applied metabolomics techniques such as NMR spectroscopy and Gas and Liquid Chromatography Mass Spectrometry (GC and LC-MS) through to more specialized technology such as electronic noses. A selection of the more common approaches will be discussed here.

### 4.1. Nuclear Magnetic Resonance (NMR) Spectroscopy

NMR spectroscopy is a powerful and well-used tool for molecular identification and structure analysis. It has been the mainstay of metabolomics/metabonomics since the terms were first defined in the late 1990s [62,63,64]. It is popular since it provides a relatively simple method for measuring the ensemble of metabolites present in a solution with minimal sample preparation and is relatively inexpensive on a per sample basis. For the interested reader, a more complete description of NMR can be found elsewhere [65,66].

NMR is primarily designed to work with samples in the solution or the solid state. This does not pose a problem for saliva samples but analysis of breath samples, especially those containing only trace amount of analytes, by NMR poses several difficulties in terms of both sample handling and in low signal-to-noise ratio of the NMR signals obtained from gases at atmospheric pressure. NMR based metabolic analysis of breath condensate has however, been carried out and a good review of the use of NMR-based metabolomics to explore airway diseases is given in Sofia et al. [67]. In such cases breath condensate, which contains predominantly water vapor but also volatile and non-volatile substances from the lower airways, is collected through spirometry or a condenser. Sample processing is minimal and usually only requires the addition to the solution of ~70 μL of deuterium oxide (D_2_O) to provide a frequency lock for the spectrometer, a standard reference compound such as sodium 3-trimethylsilyl (2,2,3,3-2H4) propionate (TSP, 1 mM), which has a chemical shift of 0 ppm, and sodium azide (3 mM) to kill any microorganisms present in the sample. Although there is a large peak in the resulting spectra from the water in the sample, this can be suppressed via several common NMR pulse sequences. Statistical analysis via techniques such as Principle Component Analysis (PCA) or Partial Least Squares (PLS) can then be used to classify the data and potentially provide diagnostic information.

It should be noted, however, that there has been some discussion of whether NMR-based metabolomic analysis of EBR is clinically useful or not [68,69]. The concern has been that the cleaning protocols used on the reusable condenser parts to clean the system, in between patients could produce an artificial metabolic fingerprint not related to the endogenous metabolic pathways under study. It would appear however that although collection devices are an important source of variability of breath condensate analysis [70,71], suitable cleaning procedures and data quality control procedures enable NMR analysis of breath condensate and saliva to be useful for metabolomics, particularly breathomic studies. Lack of full standardization of collection methods and analysis techniques does however, still hamper the introduction of such methods to routine clinical practice [72].

### 4.2. Gas and Liquid Chromatography Mass Spectrometry (GC-MS and LC-MS)

Gas Chromatography (GC) usually hyphenated with Mass Spectrometry (MS) is one of the most widely used and powerful methods within the analytical sciences. It detects and quantifies gases from 100 parts per million (ppm) to 1 part per billion (ppb) or less, and has been a mainstay of metabolomics research for many years, where it is often seen as the “gold standard” [73]. Some of the first comprehensive breath studies were performed using GC in 1971 by Pauling [74], who collected the gas from multiple breath exhalations in a cold tube then heated the tube and used gas chromatography to analyze the released gases [74]. Pauling found 250 substances in a sample of breath, and about 280 substances in a sample of urine vapor. It was thought that the new technique would be useful for medical purposes but at the time analyzing the data was too difficult and the technique did not become adopted into clinical practice. Metabolomics with its focus on combining analytical techniques, advanced statistical analysis and biological interpretation, may change this in the future.

As the name suggests samples for GC-MS must either be in the gaseous phase or, more likely, be transferred into the gaseous phase by heating. The sample is then injected into the chromatograph where an inert carrier gas (usually helium, but increasingly hydrogen) is used to transport it through a packed or open, tubular (capillary) column. The column is typically coiled and very thin (0.25 mm internal diameter) allowing even those tens of meters in length to be housed within a relatively small temperature controlled oven. The exact length of the column varies depending on the type and speed of the desired analysis but for metabolomic studies longer columns (~30 m) are used as these provide better chromatographic resolution and ensure maximum separation of the analytes. Separation of compounds occurs due to differing rates of partitioning of the components of the sample between the internal lining of the column (stationary phase) and the carrier gas (mobile phase). This means each compound exits the column at a different time (known as the retention time). The MS can then be used to detect eluting compounds, traditionally using electron impact (EI) ionization to ionize the compound, and then to measure the mass to charge ratio of each ion and generate a unique mass spectrum for the compound. SPME can also be used to absorb and pre-concentrate volatile compounds in breath prior to analysis [75,76].

Liquid chromatography mass spectrometry (LC-MS) operates on a similar principle to GC-MS except that the chromatography stage utilizes a liquid mobile phase rather than a gas. This eliminates the need for metabolite volatility so there is no requirement for sample derivatization (see below), meaning that that a much wider range of analytes can potentially be measured, the overall analysis time per sample is often much shorter than for GC-MS and a greater number of ionization mechanisms are possible. The use of liquid phase solvents also allows for a greater range of separation mechanisms than with non-reactive gases such as helium in GC. However, LC-MS can produce more variable data than GC-MS data, due to larger retention time drifts being observed.

Both GC and LC-MS offer very high chromatographic resolution and good reproducibility of results. The techniques offer increased sensitivity compared with NMR whilst extensive and easily searchable libraries of molecular fragmentation patterns facilitate metabolite identification [77]. An added bonus for breath analysis via GC is that samples are already in the gas phase and thus do not require chemical derivatization prior to analysis [78]. For saliva samples, standard chemical derivatization is still needed to make the samples volatile and thermally stable enough for GC analysis and this significantly increases the sample preparation time.

### 4.3. Capillary Electrophoresis

Electrophoresis is defined as the migration of ions under the influence of an electric field and gel electrophoresis is a commonly used method to separate proteins by size and charge [79]. The development of capillary electrophoresis (CE) was enabled by advances in GC column technology but instead of using pressurized gas or liquid as the mobile phase, CE uses high voltages to generate electrophoretic flow of ionic species within a narrow-bore (20–200 µm i.d.) capillary. CE can be linked to either an Ultraviolet–Visible spectroscopy (UV–vis) or MS detector and the resulting data look very similar to LC chromatograms; although in CE the data are referred to as an electropherogram rather than a chromatogram and retention time is referred to as migration time.

An often overlooked advantage of CE is that its sample volumes are some of the smallest of any modern separation method due to the small diameter of the typical CE capillary. Sample volumes for LC are ~20 µL, those of capillary GC are ~0.5 to 1 µL but CE sample volumes can be as low as 100, 10, and even 1 nanoliter (nL) or less. CE can also perform efficient separations of both large and small molecules making it a very versatile method.

The disadvantages of CE are that is it is tricky to set up and the narrow capillary blocks easily with buffer and salts. It is thus not as well used as other separation methods but a recent study using CE with conductometric detection used small volumes (100–200 μL) of exhaled breath to simultaneous determine inorganic cations and anions, and organic anions, such as chloride, nitrate, sulphate, lactate and potassium in patients with COPD [80].

### 4.4. NMR-GC Hyphenation

Saliva may be analyzed with NMR or GC-MS. NMR breath analysis is very difficult but would be of use in some cases; for example, to distinguish diastereomers and enantiomers which may display identical spectra at different retention times. NMR allows constitutional and configurational isomers to be distinguished but, while semi-preparative GC is an excellent technique for isolating small amounts of volatile compounds from mixtures such as breath, the recording of the NMR spectra of gases leaving a gas chromatograph which contain only trace amount of analytes imposes some problems in terms of sensitivity. Online coupling of GC with NMR has nevertheless been described for the analysis of volatile stereoisomers [81,82], caffeine [83], and menthol and menthone from peppermint oil [84]. Gas phase NMR is thus possible, although far too complex for regular use but further developments in this field are still highly desirable for breath analysis. Hyphenation of LC with NMR is also possibly but is a more specialized technique, mainly used to perform isolation and structural determination in natural product chemistry [85,86] rather than for metabolomics studies.

### 4.5. Direct Injection Mass Spectrometry

There are various forms of direct injection MS that have been used for the analysis of trace amounts of VOCs such as acetone, acetaldehyde, methanol, ethanol, benzene, toluene, xylene and inorganic gases in air and breath [87]. Such methods include secondary electrospray ionization-mass spectrometry (SESI-MS) [88], proton-transfer-reaction mass spectrometry (PTR-MS) [89] and selected ion flow tube mass spectrometry (SIFT-MS) [90].

The SESI-MS method is not so well used but both PTR-MS and SIFT-MS are popular methods of analysis in a range of areas including environmental, food and health sciences [89,90]. In PTR-MS, a hollow cathode ion source produces H_3_O^+^ ions from high purity (>99%) distilled water. The reagent ions enter a drift tube where the trace compounds are ionized either via proton transfer (H_3_O^+^) before analysis with either a quadrupole or a high-resolution time-of-flight mass analyzer. A disadvantage of PTR-MS is that is can only work for target molecules with a proton affinity higher than that of water. SIFT-MS is similar to PTR-MS but uses a greater number of precursor ions for chemical ionization (H_3_O^+^, NO^+^, or O_2_^+^) and thus works with a wider range of exhaled metabolites. Both systems allow real-time, quantitative analysis and eliminate the need for sample preparation, pre-concentration and chromatography or other forms of separation. They do not, however, detect as many compounds as other forms of analysis such as GC and LC.

### 4.6. Ion-Mobility Spectrometry

Ion-mobility spectrometry (IMS) is an analytical technique used to separate and identify ionized molecules in the gas phase based on their mobility in a carrier buffer gas, and when coupled with MS (e.g., IMS-MS), enables additional separation of ions by their mass-to-charge ratio [91]. Furthermore, there are few variants of IMS that enable the use of an external electric field at ambient pressure and temperatures in order to separate different ions formed from the target analytes; these include differential mobility spectrometers (DMS) and high-field asymmetric waveform ion mobility spectrometers (FAIMS) [10]. Generally, IMS is commonly used to detect explosives, chemical warfare agents or illegal drugs but more recently has been used to analyze VOCs in breathomic studies [92]. Typically, VOC detection limits using IMS are of the magnitude of pg/L to ng/L-range, and when IMS is coupled with a multi-capillary column as a breath sampling device, the analysis can be performed in under 8 min and at the site of sampling [93].

### 4.7. Ultra Violet and Infra Red Spectroscopy

Spectroscopy is based on the measurement of absorption of electromagnetic radiation by a compound, or compounds of interest. Spectral fingerprints of compounds in breath span the UV to the mid-IR spectral regions. Typically, exhaled breath samples require pre-concentration prior to analysis via SPME, a suitable absorbent material or by direct cryofocusing [94]. Compounds present in exhaled breath that are IR or UV active such as ammonia, carbon monoxide, carbon dioxide, methane and ethane absorb light at wavelengths characteristic of the bonds present in the molecule and these absorption bands can be used to identify specific molecular components and/or to allow identification of a compound via reference library matching. Stable isotopomers of IR active molecules can also be accurately detected, making it possible to follow specific metabolic processes. While infrared spectroscopy data are not as detailed as those from NMR or MS based methods the technique has the advantages that it is quick, simple, non-destructive and does not require extensive sample preparation; near real-time data can be obtained and the instruments are much lower in cost that NMR or MS instruments. The disadvantages are that spectroscopy is not as sensitive or selective as MS, with detection limits in the ppm to ppb range and the technique is also limited in the number of chemical species it can distinguish.

Although specialized methods such as cavity ring-down spectroscopy (in which light is trapped for several microseconds in between two highly reflective mirrors) can increase the sensitivity of spectroscopic techniques these methods are not widely used and still do not detect as wide range of compounds as MS. Breath analysis using laser spectroscopic techniques is only a very recent advancement and, to a great extent relies on recent development of diode lasers capable of covering a wide spectral range of molecular fingerprints [95]. A good review of breath analysis using laser spectroscopic techniques is given in [95,96].

### 4.8. Electronic Noses

Electronic noses (E-noses) are artificial sensor systems, usually consisting of a range of sensors for various chemicals of interest. E-noses are able to detect (‘*smell*’) patterns of VOCs in breath and then use algorithms for classification of the ‘*breathprint*’ and comparison with previously recorded samples from known sources. Such methods can be paired with, and add value to existing diagnostic tests, such as routine spirometry [97]. Although a relatively new technique, e-noses have been used to discriminate between patients with respiratory disease, including asthma, COPD and lung cancer, and healthy control subjects, and also among patients with different respiratory diseases and with airway inflammation activity [98].

### 4.9. Data Processing and Statitical Analysis

Data processing typically consists a series of computational steps that are used to convert the complex raw data generated by the analytical instruments into a usable and ‘human readable’ data format, which can then be used for further statistical analysis. Several statistical methods are used to understand the overall behavior of the different biological systems/conditions under consideration as well the contribution of each metabolite to any differences between the systems/conditions. Multivariate methods such as PCA and Discriminant Analysis methods (PLS-DA, O (orthonganal) PLS-DA) are used for the analysis of all the metabolites that are measured in the biological systems. Clustering analysis methods such as Hierarchical Clustering Algorithm (HCA) and partitioning clustering methods (K-means, self-organizing maps (SOMs)) are used to cluster similar samples or similarly behaving metabolites. Univariate analysis methods such as the t-test, Mann-Whitney test and the different ANOVA methods are used to investigate whether a specific metabolite is significantly different between the different biological systems/conditions. Since a univariate analysis would need be performed for each metabolite studied, the results need to be corrected for false positives using methods such as the Bonferroni correction and Benjamini-Hochberg correction algorithms [99].

## 5. Breathomics and Salivaomics Applications

While the total number of saliva and breath related metabolomics-based studies are limited when compared to all metabolomics-based studies, there is still a wide breadth of salivaomics and breathomics research related to disease diagnostics and characterization. The following section provides a few applications and the potential of this technology for the development of rapid diagnostic methods and personalized medicine [1].

### 5.1. Infectious Diseases

Owing to the complexity of the salivary microbiota and their related metabolome, and the VOCs expelled through breath, salivaomics and breathomics can inform on the different stages of infection (i.e., early-to-intermediate), allowing more time for diagnosis and treatment of infections. A recent GC-MS and LC-MS based approach, for example, demonstrated the use of saliva to detect gingivitis (generally caused by *Porphyromonas gingivalis*) and other periodontal infections [11]. The combination of LC-MS and GC-MS was able to identify cellular level stress, degradation of purines and glutathione (by oxidative stress) and increased activities in amino and fatty acid synthesis pathways [11]. There has also been progress in the detection of infectious diseases with higher social impacts such as tuberculosis, especially in developing countries. A recent study utilized the conversion of isotopic ^13^CO to ^13^CO_2_ by the pathogen *Mycobacterium bovis* in rabbit populations. The testing indicated that detection of CO_2_ generated through the activity of mycobacterial CO dehydrogenase (CODH) had the potential to provide rapid and non-invasive diagnosis of tuberculosis [100]. While the study by Maiga et al. [100] demonstrated the preclinical breath analysis of *Mycobacterium bovis* in rabbit populations, if developed further it could enable for cheaper and faster point-of-care diagnosis of tuberculosis within a clinical setting which would augment and improve current pathology tests. Zhu et al. [101] extended breath single biomarker analysis further and used the entire ‘*breathprint’* to diagnose acute *Pseudomonas aeruginosa* and *Staphylococcus aureus* lung infections in a mouse model. Such an approach that uses the entire ‘*breathprint*’ rather than single biomarkers enables volatile metabolites to be characterized and monitored during the course of infection, and potentially identify a suite of breath biomarkers that can be used in the diagnoses of pathogens at any point during the infection [88,101,102].

**Figure 1:**
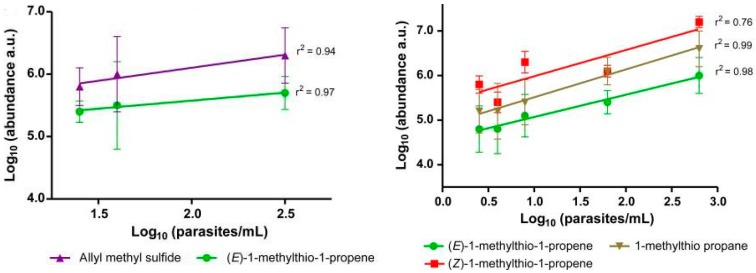
Correlation of identified malaria metabolites with parasite level in exhaled breath before and after antimalarial treatment with a fast-acting synthetic ozonide drug. Error bars show the standard deviation of the mean. (After Berna et al. [22]).

A recent study by Berna et al. [22] investigated the relationship between *Plasmodium falciparum* (the parasite that causes malaria) and the exhaled breath of individuals infected with the parasite within a controlled clinical study. The current approach for malarial diagnosis involves an assessment of the patient’s clinical presentation and an analysis of a sample of blood via microscopy; which is both time consuming and expensive. In the study by Berna et al., breath samples were collected using sorbent tubes and analyzed via GC-MS to detect specific malaria-associated VOCs. In total, 9 compounds were identified and their concentrations varied significantly over the course of the infection. The identified metabolites comprised: carbon dioxide, isoprene, acetone, benzene, cyclohexanone, allyl methyl sulfide, 1-methylthio-propane, (*Z*)-1-methylthio-1-propene, and (*E*)-1-methylthio-1-propene [22]. Figure 1 illustrates the correlation of these identified metabolites with the parasite level within the patients sampled.

If the work could be extended and the biomarkers shown to be unambiguously related to malaria infection, the method could be used to screen patients’ breath for the presence of malaria, which would be a rapid and cost effective tool in developing countries where access to medical treatment is both geographically difficult and cost limited.

### 5.2. Influenza Infection and Possible Antiviral Applications

Influenza is a highly contagious respiratory disease that causes high global morbidity and mortality [103]. Understanding the pathogenesis of influenza virus is: critical for effective disease control in a pandemic scenario, enables the screening of the emergence of new strains with pandemic potential, and facilitates the development of vaccines and antiviral drugs. The application of metabolomics to study the dynamics of influenza infection with host metabolism is in its infancy, with the majority of work to date being done using viruses grown in cell cultures [104,105,106,107,108,109]. However, Aksenov et al. examined VOCs produced directly at the cellular level from B lymphoblastoid cells upon infection with three influenza A virus subtypes (H9N2 (avian), H6N2 (avian), and H1N1 (human)) [110] via headspace GC-MS. The patterns of VOCs produced in response to infection were unique for each subtype and the metabolic flux of the VOC released post infection were different. The emitted VOCs included esters and other oxygenated compounds, which was attributed to the increased oxidative stress resulting from the viral infection. It was concluded that elucidating such VOC signatures from the host cells response to infection may yield non-invasive diagnostics of influenza and other viral infections [110]; this approach has been applied for the determination of *Plasmodium falciparum* (malaria) in humans [111] and *Acinetobacter baumannii* colonization in the lower respiratory tract (which results in ventilator-associated pneumonia in patients admitted to hospital) [112].

Progressing from in vitro studies, animal models of influenza infection such as the mouse, have been used to study metabolic changes associated with influenza infection at an organism level [113,114]. In mice, influenza infection was associated with both systemic (serum) and localized (lung and Broncho-alveolar lavage) changes of more than 100 metabolites that were associated with the pulmonary surfactant system, suggesting viral-induced lung injury [114]. A metabolome-wide association study with cytokines using high resolution metabolomics in the influenza-infected mouse lung also revealed high correlation of 396 metabolites with pro-inflammatory cytokines, such as IFNγ, IL-1β, TNF-α and anti-inflammatory cytokines, such as IL-10 [113].

In humans, metabolomics has been applied as a diagnostic tool for acute respiratory distress syndrome caused by influenza A (H1N1) infection via NMR analysis of collected blood serum [115]. Using GC-MS analysis of the breath collected from individuals that were vaccinated with live attenuated influenza, Philips et al. reported a distinct VOC signature in the vaccinated group showing the potential to use breathomics to diagnose influenza infection [116]. However, the focus of targeting other important sampling sites, such as saliva or the upper respiratory tract where the viral replication occurs is well less reported. Besides clinical trials, an alternative to the investigation of the application of salivaomics and breathomics in influenza infection is the animal model of influenza infection. Among the different animal models of influenza infection, sampling from the breath, saliva and nasal cavity can only be done in larger animals such as the ferret [117,118]. To date, there are limited metabolomics studies in ferrets, however, with the increasing use of this model to study different aspect of influenza pathogenesis [117]. This is because ferrets are considered to be the ‘gold standard’ animal model in influenza research due to the fact that they are susceptible to infection with human influenza viruses and display similar clinical symptoms [119], Therefore, it can be anticipated that, with the increasing use of this model to study different aspect of influenza pathogenesis [117,120], the study of metabolomics in ferrets that includes saliva, breath and/or nasal wash, can prove to be valuable.

### 5.3. Cancer and Degenerative Diseases

Ishikawa et al. [26] investigated the suitability of salivary biomarkers for the determination of oral cancer using isotope-labeled CE-TOF-MS. In this study, 85 metabolites were observed to show significant differences between tumor and matched control samples, with a subset of metabolites subsequently validated in tissue-based comparisons. It was concluded that integrating both saliva and tumor tissue metabolomics eliminated false biomarker discovery that are coincidentally different between oral cancers and controls. Through the application of similar comprehensive biologically matched sampling, the identification of saliva biomarkers could be the basis of a clinically feasible method of non-invasive oral cancer screening [26]. CE-TOF-MS was similarly used to determine salivary biomarkers in Yusho patients [121]. 

Mueller et al. [122] developed and validated a GC-TOF-MS method investigating the analysis of saliva from smokers and non-smokers. The method was applied to saliva of smokers and non-smokers within a 24 h diet-controlled clinical study in order to identify biomarkers that could be linked to smoking related diseases. In total, 13 metabolites were identified, of which metabolites such as tyramine, adenosine, and glucose-6-phosphate, which were linked to smoking-related biological perturbations [122].

Breath testing techniques such as electronic nose devices have been used to detect and differentiate the patients suffering from Alzheimer’s and Parkinson’s diseases [123]. The study used an e-nose (for VOC sampling) in conjugation with ion mobility spectrometry. The sample analysis was confirmed by use of an existing standard enzyme-linked immunosorbent assay (ELISA) technique for the detection of the possible causative agent, amyloid beta (Aβ). The predictability of the conducted tests to detect Alzheimer’s and Parkinson’s diseases was reported to be ca. 94%. Likewise, other chronic degenerative diseases such as liver cirrhosis can be detected at a comparatively early stage by VOC sample analysis [124]. The experiment involved the detection of signature metabolome profiles for chronic liver disease (CLD) and compensated cirrhosis (CIR). GC-MS analysis involved the collected exhaled breath in Tedlar^®^ collection bags (E. I. du Pont de Nemours and Company, Wilmington, DE, USA). Approximately 11 discriminatory metabolites were observed for CLD and CIR. These metabolites comprised propanoic acid, octane, terpene, dimethyl sulphoxide (increased), methylbutanal and hexadecanol (depleted), possibly caused by impaired cytochrome P450 detoxification mechanisms in the liver [124].

Lung cancer studies has also been one of the areas where the breath-based metabolomics has been extensively applied. In one of these studies, selective alkanes, aldehydes and other metabolites such as acetone have been reported as biomarkers for lung cancer. However, in almost all cases, the prediction in terms of analysis sensitivity and specificity of these tests have been limited [125]. Recent research conducted by Peralbo-Molina et al. [126] involved the analysis of breath samples from smoking, non-smoking and past smoking patients in order to detect the metabolic signature within exhaled breath condensate for risk and cancer affected individuals. In addition to the previously detected alkanes [125], a high resolution GC-TOF-MS approach was able to identify monoglycerols and squalene as the prominent signature metabolites. Although the sensitivity and specificity for these tests were noticeably higher than the previously reported values, sensitivity was still below 85%, although, in the case of a distal airway metabolite analysis, the specificity of about 90% was observed.

Pointing to the limitations of breath analysis, it has also been suggested that using breathomics in combination with cellular sampling/cell culture metabolomics to order to increase the sensitivity, specificity and, as a result, the predictability of the cancer diagnostic metabolomics. In a recent review, the small number of signature metabolites was identified as one of the major limitations of ‘breath only’ analyses. Kalluri et al. [127] suggested that metabolic profiling of cell cultures and breath analysis be used in parallel to determine the signature metabolites attributed to lung cancer. The study indicates the origin of previously indicated signature metabolites from both healthy and affected individuals; the metabolites include aldehydes (formaldehyde, acetaldehyde), ketones (acetone) and various alkanes, which are formed during cellular oxidative stress, as observed during lung cancer. In such cases, cellular hypoxia has been observed, where sugars are metabolized in absence of oxygen via glycolysis only and the Krebs cycle remains unutilized. Secretion of the abovementioned metabolites in addition to others such as excessive lactic acid (leading to cellular autophagy) can be used as more reliable indicators of lung cancer as compared to standalone breathomics. For a detailed overview of cancer signature metabolites, the reader is suggested to refer to a recent review by Patel and Ahmed [128].

### 5.4. Respiratory Disorders

Asthma is a widespread disease with global estimates suggesting as many as 334 million people are affected. Asthma causes significant socioeconomic impacts with growing direct health-related costs of treating and managing affected people and in-direct costs relating to lost productivity (absence from school and work) [129]. Current approaches used to diagnose and monitor asthma include a physical examination and a range of lung function tests, allergy testing, exhaled nitric oxide (ENO) tests, imaging, sputum eosinophils (count of white blood cells in sputum (saliva and mucus), and provocative testing (exercise and cold-induced asthma). The time for diagnosis can be rather drawn out and, as such, metabolomics provides a unique opportunity where molecular determinants can be used to rapidly diagnose asthma and other respiratory illnesses [130,131]. To date, medical metabolomics studies have been limited in size and scope, with a large proportion of research focused on quantifying the small biochemicals in plasma and tissue samples in order to identify biomarkers that may serve as therapeutic targets [132]. In more recent times, the focus for metabolomics-based research has been on using breath samples, amongst other non-invasive sample matrices, to diagnose asthma from other respiratory illnesses amongst children [133].

In a study by Carraro et al., NMR-based metabolomic analyses of exhaled breath condensate was used to discriminate between asthmatic and healthy children within a pediatric clinical setting [6]. Twenty-five children with asthma and 11 age-matched healthy control children were recruited in the study. Each child provided a breath condensate sample using an EBC condenser over a period of 15 min. The collected samples were then dried prior to reconstituting in D_2_O for NMR analysis. In addition to the EBC sample, the children were tested using conventional asthma techniques such as ENO and measuring lung function via spirometry. Typically, the combination of ENO and lung function testing discriminates children with asthma and healthy children with a success rate of ca. 80%, whereas selected signals from NMR spectra offer a slightly better discrimination (ca. 86%). The selected NMR variables derive from the region of 3.2–3.4 ppm, indicative of oxidized compounds, and from the region of 1.7 to 2.2 ppm, indicative of acetylated compounds [6]. In a similar study, 57 asthmatics and 22 healthy controls were recruited and EBC samples collected and analyzed by NMR. Thirteen spectral regions were identified as for discriminating between asthmatics and the healthy control cohort. The normalized peak areas were used in multivariate logistic regression, and a model consisting of five regions predicted the asthmatics (82.3%) [49]. It is noted that the authors did not attempt to identify the compounds in the discriminating spectral regions. Motta et al. investigated two different condenser trap temperatures (−4 °C and −37 °C) and evaluated the effect using NMR [134]. It was found that the two different temperatures resulted in no significant variation between the analyzed EBC when analyzed separately and as part of a blind analysis.

Pulmonary arterial hypertension (PAH) is a life-threatening condition in which the patient deteriorates over time. PAH is defined as where a patient has high blood pressure in the pulmonary arteries that go from the heart to the lungs [135]. This is a result of the small arteries in the lungs becoming narrow or blocked, which in turn increases the blood pressure in the lungs and puts stress on the heart as it works harder to pump blood to the lungs. Congestive heart failure, blood clots in the lungs, HIV, drug abuse, liver disease, autoimmune diseases, heart defects, lung diseases and sleep apnea have all been linked to PAH [135]. Current approaches used to diagnose PAH includes a physical examination and a range of specialist tests, such as: echocardiogram (ultrasound), computed tomography (CT) scan, ventilation-perfusion scan (to identify blood clots), electrocardiogram, chest X-ray and provocative testing (exercise).

**Figure 2:**
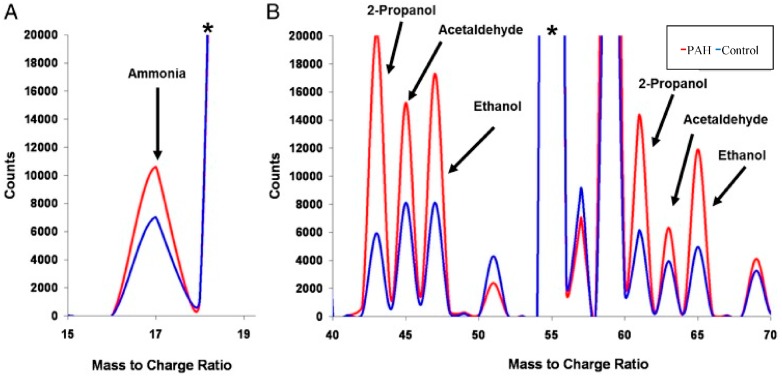
Representative selected ion flow tube mass spectrometry (SIFT-MS) chromatogram for the pulmonary arterial hypertension (PAH) and control cohort groups [137]. Labeled peaks correspond to compounds that were significantly different in patients with PAH compared to the control cohort. (**A**) Ammonia peak comes from the O_2_^+^ precursor ion spectrum; (**B**) Other molecules peaks from the H_3_O^+^ precursor ion spectrum. * Precursor ion peak. (Reprinted from Chest, 145 (3), Cikach, F.S., A.R. Tonelli, J. Barnes, K. Paschke, J. Newman, D. Grove, L. Dababneh, S. Wang, and R.A. Dweik, Breath Analysis in Pulmonary Arterial Hypertension, pp. 551–558, Copyright (2014), with permission from Elsevier).

Zhao et al. [136] investigated the application of metabolomics as a diagnostic tool in human lung tissue samples collected from patients diagnosed with PAH and age-matched controls. Using a combination of LC-MS and GC-MS, it was identified that patients diagnosed with PAH showed unbiased metabolomic profiles of disrupted glycolysis, increased tricarboxylic acid (TCA) cycle, and fatty acid metabolites with altered oxidation pathways; indicating increased Adenosine triphosphate (ATP) synthesis. It was concluded that these biomarkers could be used for the diagnosis of PAH, however, collecting lung tissue samples is considered invasive. An alternative approach would be to analyze the exhaled breath of patients diagnosed with PAH. Such an approach was undertaken by Cikach et al. [137], where fasting state breath samples were collected and analyzed by SIFT-MS. It was found that the concentrations of the exhaled ammonia, 2-propanol, acetaldehyde, ammonia, ethanol, pentane, 1-decene, 1-octene, and 2-nonene were significantly different in patients with PAH compared to the control cohort (Figure 2) [137], with the concentration of compounds correlating with the severity of PAH. This suggests that differences in the breath metabolic profile can potentially be used to diagnose and classify the severity of PAH. Furthermore, with such observed differences, there is potential for the development of rapid diagnostic breath analyzers that could be used to monitor PAH progression. Such metabolomic approaches and tool development are also being applied to other respiratory diseases, such as acute respiratory distress syndrome (ARDS) [138,139], chronic obstructive pulmonary disease (COPD) [52,140], lung cancer [126], or diseases with a clinically relevant respiratory component including cystic fibrosis [141,142] and primary ciliary dyskinesia [143,144].

## 6. Future Direction and Conclusions

In many fields of medicine, there is a growing interest in characterizing diseases at the molecular level with a view to developing an individually tailored therapeutic approach. A comprehensive metabolome analysis, using a range of invasive and non-invasive sample methodologies such as saliva and exhaled breath would result in a greater depth of knowledge being obtained for specific diseases and disorders. Such an approach would enable the tracking of metabolic pathways, monitor the effectiveness of therapeutic treatments and interventions, and assess their impact on the entire biological system.

Through the application of breathomics and salivaomics techniques specifically, it is envisaged that biomarkers will ultimately be identified in saliva and breath samples that are specific to a range of oral and respiratory diseases and disorders, in addition to other systemic health conditions. Through such biomarker discover, and validation using traditional diagnostic methods, it is anticipated that a range of rapid diagnostic methods such as colorimetric assays and targeted metabolite breathalyzer and saliva swab technologies will be developed. Such biomarker discovery and assay/technologies can be used to develop rapid test kits for disease diagnoses that can be used in clinics to identify specific infections and diseases (such as malaria, PAH, cancer etc.) and enable medical interventions to be started earlier.

Furthermore, breath analysis is a tool which can potentially be used for human exposure assessment. Although efforts have been made to optimize breath analysis methods, there is still a need for more studies before these methods can be used routinely. These studies should involve the standardization of collection methods, and profiling via the various detection platforms. Multiple efficient devices have also been developed which have shown promise, however, there are still issues involving leakage, adsorption and transfer processes. Lastly, the use of more sensitive and portable methods should allow for more accurate identification and quantitation within a clinical environment.

Saliva, like breath, has more recently been accepted as an important diagnostic fluid (along with blood and urine). This has been due to the significant improvements achieved with instrumentation, particularly sensitivity, which is needed to analyze it. Although there has been a great advancement in instrumentation, there is still a need to establish even more sensitive detection methods, measure circadian variations in saliva, and better define the biological correlations between saliva and other biofluids. This review suggests that specific diagnostic uses of saliva hold considerable promise, however much of this (like breath), depends on further studies. Further work could involve the use of saliva in monitoring immune responses to infection, as well as therapeutic drug levels and the detection of use of illegal drugs. In addition to this, pilot studies should be confirmed in larger, controlled trials.

Another advantageous non-invasive biological fluid for future consideration in metabolomics research is sweat. The discovery of sweat biomarkers could be used in a transdermal patch, where the patch will monitor an infection based on the unique identified biomarker(s) present that relate to specific disease/infection (and their effective treatment). Such technologies for rapid diagnostics and therapy monitoring will have the greatest impact where access to health care services (clinics and pathology labs) is limited or not available (such as rural and remote indigenous communities and developing counties).

## Figures and Tables

**Table 1:** Breath sample collection devices and their characteristics.

Breath Type	Characteristics		
	Collection Device	Advantages	Disadvantages
Initial	Tedlar bags Sampling tube/bulb Tenax cartridges	Simple Simple Relatively simple	Losses of sample, possible contamination, unstable over time Time-consuming Potential for contamination
Modified (alveolar breath)	Adsorption tube Haldane-Priestly tube Bio-VOC sampler EBC condenser ReCIVA sampler RTube sampler QuinTron sampler	Losses are controlled Simple to use and portable Preservation of original sample Commercially available Commercially available, repeatable and reproducible, storage on sorbent tubes Commercially available, single use design, handheld device Commercially available, disposable, home testing	Poor efficiency Possible adsorption capability No control of breathing and CO_2_ level Further validation needed Further validation needed Further validation needed Further validation needed

## Abbreviations

ATP	Adenosine triphosphate
CE	Capillary electrophoresis
CIR	Compensated cirrhosis
CKD	Chronic kidney disease
CLD	Chronic liver disease
COPD	Chronic obstructive pulmonary disease
CT scan	Computed tomography scan
CV	Coefficient of variability
D_2_O	Deuterium oxide
EBC	Exhaled breath condensate
EBV	Exhaled breath vapor
EEI	Extractive electrospray ionization
EI	Electron impact
ELISA	Enzyme-linked immunosorbent assay
ENO	Exhaled nitric acid
FID	free induction decay
fMRI	Functional magnetic resonance imaging
GC	Gas chromatography
HCA	Hierarchical clustering algorithm
IBS	Irritable bowel syndrome
IR	Infrared
LC	Liquid chromatography
MS	Mass spectrometry
MS	Mass spectrometry
NMR	Nuclear magnetic resonance spectroscopy
OPLS-DA	Orthogonal partial least squares-discriminate analysis
PAH	Pulmonary arterial hypertension
PCA	Principle component analysis
PLS	Partial least squares
PLS-DA	Partial least squares-discriminate analysis
PTR	Proton-transfer-reaction
SESI	Secondary electrospray Ionization
SIFT	Selected ion flow tube
SPME	Solid phase microextraction
TCA	Tricarboxylic acid
TOF	Time-of-flight
UHPLC	Ultra-high performance liquid chromatography
UV	Ultraviolet
UV–vis	Ultraviolet–visible spectroscopy
VOCs	Volatile organic compounds
